# First Reported Case of Lesser Sac Empyema Secondary to Foreign Body Perforation

**DOI:** 10.7759/cureus.47186

**Published:** 2023-10-17

**Authors:** Yat Cheung Chung, Binura Lekamalage, Ashray Rajagopalan, Asiri Arachchi

**Affiliations:** 1 Department of General Surgery, Monash Health, Melbourne, AUS; 2 Department of General Surgery, Tauranga Hospital, Bay of Plenty, NZL; 3 Department of Colorectal Surgery, Monash Health, Dandenong, AUS

**Keywords:** surgical endoscopy, lesser sac empyema, foreign body, case report, general surgery

## Abstract

This is the first reported case of lesser sac empyema secondary to a foreign body perforation in the posterior stomach. Although PubMed and Google Scholar search reports cases of lesser sac empyema alone and foreign body penetrations, there are currently no reported cases of a lesser sac abscess secondary to a foreign body. Patients with a lesser sac empyema present atypically with an insidious onset. The lesser sac should be examined in patients with peritonitis without a clear source.

A 48-year-old female presented to the emergency department with acute onset epigastric pain. The patient was tender in the epigastrium and left upper quadrant with associated guarding. The patient had elevated white cell count and C-reactive protein, with a computed tomography scan identifying a foreign body posterior gastric wall perforation. The patient was managed with endoscopic drainage of the lesser sac empyema and surgical washout of the abdomen.

Foreign bodies are investigated using different imaging modalities, with computed tomography being able to further evaluate the size, shape, and complications. Intra-abdominal collections can be managed through three different methods: percutaneous drainage, endoscopic drainage, and surgery. Patients with peritonitis would require a laparoscopic or open surgical washout of the abdomen and inspection of the lesser sac would be necessary if no obvious source is identified.

Foreign body ingestion requires careful history taking and assessment. Patients with lesser sac empyema present atypically, and this can lead to delayed surgical referral and management. Contained intra-abdominal collections can be drained percutaneously or endoscopically.

## Introduction

Foreign body (FB) ingestion is rare in adults compared to children [1]. Of FB ingestions, 95% are related to food, such as fish and chicken bones, and toothpicks [2]. These tend to become impacted in the physiological narrowings of the oesophagus at the upper oesophageal sphincter, at the level of the arch of the aorta, and the diaphragmatic hiatus.

Patients with intestinal perforation by an ingested FB often present with an acute abdomen, fever, peritonitis, nausea, and vomiting. These often occur in the elderly, alcoholics, psychiatric patients, and patients who wear dentures [3].

Plain radiographs may not identify some objects such as wood, thin bones, plastic, and glass, so computed tomography of the abdomen and pelvis (CTAP) is recommended in cases of suspected perforation secondary to FB ingestion. CTAP can also evaluate the FB in terms of size, shape, location, and depth of penetration to guide management.

Although FB ingestion and associated perforation are common, there are currently no reported cases of lesser sac empyema formation following FB perforation. Lesser sac empyema can occur from the perforation of the posterior gastric fundus or gastric body. Patients with lesser sac empyema usually have an atypical presentation, due to the contained perforation, though this may develop into generalised peritonitis.

An FB within the stomach and duodenum should be retrieved within 24 hours if it is sharp, magnetic, or greater than 5 cm in length, and within 72 hours if the diameter is greater than 2 cm or batteries that are not passed within 24 hours [4].

If the empyema is contained, it can be drained endoscopically. However, patients who present with peritonitis should proceed directly to surgery for a washout of the abdomen.

## Case presentation

A 48-year-old female presented to the emergency department with acute epigastric pain, on a background of peptic ulcer disease on pantoprazole, smoking, and occasional alcohol use. On examination, the patient was tender in the epigastrium and left upper quadrant with guarding. On full blood examination, she had leucocytosis (15.6 x 10^9/L) and had a C-reactive protein level of 434 mg/L. Erect chest X-ray and electrocardiogram were unremarkable.

**Figure 1:**
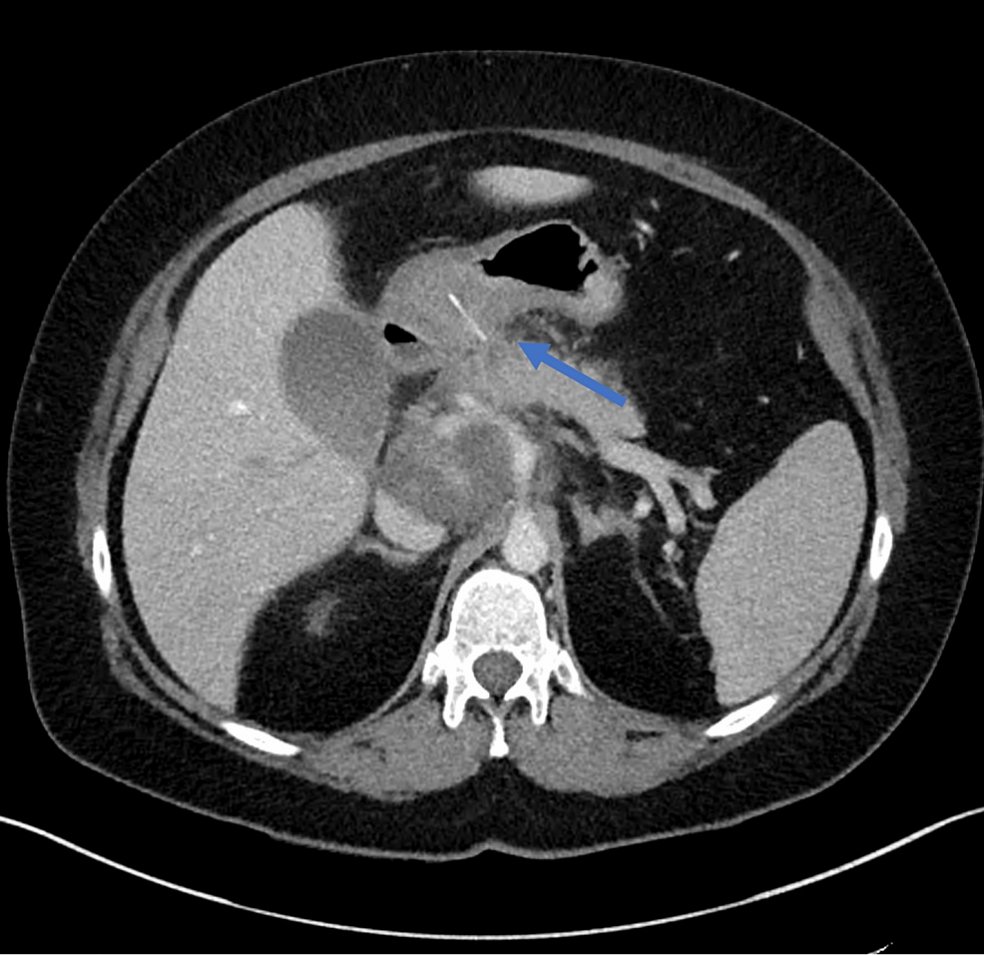
CT of the abdomen and pelvis with portal venous contrast axial view on initial patient presentation. A foreign body is indicated by the arrow.

A CTAP was performed and demonstrated a likely ulcer or partial FB perforation on the posterior wall of the gastric antrum, with regional lymphadenopathy (Figure 1). The patient proceeded to gastroscopy, which revealed pus in the inferior pyloric region without an obvious FB. The patient was started on intravenous antibiotics (piperacillin/tazobactam) and pantoprazole.

**Figure 2:**
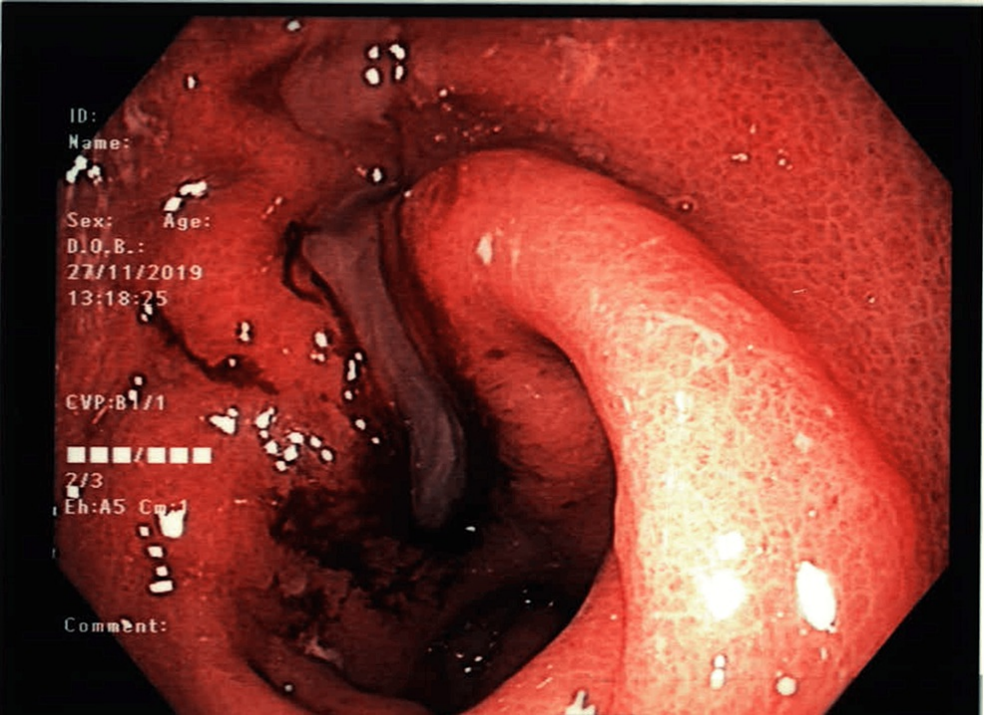
Patient’s second gastroscopy showing perforation of the posterior pre-pyloric region with pus entering the stomach.

Two days after the gastroscopy, the patient developed severe abdominal pain and proceeded to a repeat gastroscopy, which demonstrated a large amount of pus within the stomach. A pin-hole-sized perforation was identified in the posterior pre-pyloric region (Figure 2). The site of perforation was dilated to 10 mm using a balloon and the gastroscope was advanced into the cavity. A piece of chicken bone was retrieved and a 7 Fr pigtail cystogastrostomy drain was left in situ to help facilitate drainage of collection.

Following the second gastroscopy, the patient became febrile and tachycardic and proceeded to the theatre for laparoscopy. There was free fluid in all four quadrants and a large collection of pus in the lesser sac. The lesser sac was carefully entered using a lateral approach (through the gastrosplenic ligament), and a washout of both the lesser sac and all four quadrants was completed laparoscopically. Jackson-Pratt drains were placed within the lesser sac cavity and anterior to the stomach. The patient was subsequently transferred to the ICU.

The patient’s post-operative course was complicated by a portal vein thrombus, which was incidentally diagnosed on follow-up CTAP the day after surgery. The patient was gradually uptitrated to therapeutic anticoagulation, and the pigtail drain was removed endoscopically 10 days post-operatively.

## Discussion

Foreign body ingestion

FB ingestion is rare in adults compared to children [1]. FBs are usually impacted at the physiological narrowings; however, most ingested foreign bodies will pass spontaneously, although 10-20% will require removal by the endoscopic intervention [5]. Food products represent 95% of FB found in the oesophagus such as fish bones and chicken bones [2]. In this scenario, the FB had traversed the oesophagus and impaled the posterior wall of the stomach into the lesser sac.

Patients with perforation by an ingested FB are rare and most often present with an acute abdomen, fever, peritonitis, nausea, and vomiting. This is most common in the elderly, alcoholics, psychiatric patients, and patients who wear dentures [3].

Imaging can aid in the initial evaluation of the FB perforation. However, imaging should not delay definitive management, especially in patients with obstructive symptoms, such as drooling and difficulty swallowing. Typically, erect chest X-rays and abdominal X-rays can identify radio-opaque objects; however, they may not detect other foreign bodies such as wood, thin bones, plastic, or glass. Computed tomography (CT) scan is the preferred imaging modality in cases of suspected FB perforation. Studies have shown that CT scans have a sensitivity and specificity of 85.7-100% and 66.7-100%, respectively. It can evaluate the FB in terms of size, shape, location, depth of penetration, number, and complications from the FB [6-8].

FB retrieval is typically performed endoscopically and very rarely surgically. Complete oesophageal obstruction, battery ingestion, or sharp FB in the oesophagus should be retrieved endoscopically within two hours ideally, up to six hours. Patients with incomplete oesophageal obstructions can be retrieved within up to 24 hours [9-11]. FB within the stomach and duodenum should be retrieved within 24 hours if it is sharp, magnetic, or greater than 5 cm in length, and within 72 hours if the diameter is greater than 2 cm or batteries that are not passed within 24 hours. FBs that are not sharp or magnetic with a diameter of less than 2.5 cm and less than 5 cm in length can be managed conservatively and be expected to pass within the first two days. Further endoscopic evaluation is recommended if the FB is still within the stomach after three to four weeks or within the duodenum after one week. Surgery would be indicated if the patient develops complications such as obstruction or perforation or for non-progression of a FB radiographically for three consecutive days.

Lesser sac empyema

Lesser sac empyema secondary to FB perforation of the gastrointestinal tract has not been previously reported. Since posterior gastric perforation can lead to contained empyema within the lesser sac, these patients may initially present atypically. Early recognition of this differential diagnosis is important to avoid delays to surgical referral and management, as in our presented case [12].

Although intra-abdominal collections are commonly managed with percutaneous drainage [12], the lesser sac is challenging to access via this approach. The endoscopic approach is an alternative, with the assistance of endoscopic ultrasound. This provides several advantages, including clear images, identification of blood vessels in Doppler mode, and real-time images during the procedure [13,14]. A drain or stent can also be inserted using this technique.

Patients proceeding to surgery may require laparoscopic or open washout of the abdomen. Posterior gastric perforation can be difficult to identify intraoperatively, and so examination of the lesser sac may be necessary by Kocherization of the duodenum.

## Conclusions

FB ingestion is rare in adults and a careful history is required to help diagnose the cause of presentation. Patients with lesser sac empyema may present atypically, and recognition of this important differential diagnosis is necessary to facilitate prompt surgical referral and management. Contained intra-abdominal collections can be drained percutaneously or endoscopically. However, patients with peritonitis will require surgical washout of the abdomen, and the lesser sac should be assessed if no obvious culprit is found.
